# Striving for Personal Perfection: Rudolf Allers’s Psycho-Ethic-Metaphysical Approach to Character and Self-Improvement

**DOI:** 10.1007/s10943-022-01605-6

**Published:** 2022-07-05

**Authors:** Joaquín García-Alandete

**Affiliations:** Faculty of Psychology and Speech Therapy, Department of Personality, Evaluation and Psychological Treatments, University of Valencia, Avda. Blasco Ibáñez, 21, 46010 Valencia, Spain

**Keywords:** Rudolf Allers, Character, Self-improvement, Psycho-ethic-metaphysical approach

## Abstract

The Viennese psychiatrist Rudolf Allers was one of the principal authors that studied character and contributed to understanding its development and education, including the neurotic character. His psychological observations were based on his own clinical experience, his individual psychology, phenomenological and existential philosophies, and, above all, the doctrine of the Fathers of the Catholic Church and Thomas Aquinas. This paper presents Allers’s main ideas about self-improvement as a process of personal changing toward self-perfection, that is, toward the best version of oneself. For Allers, self-perfection implies the modification of insane aspects of character such as egocentricity, pride, and untidy love for oneself, which are the most important impediments to self-improvement.

## Introduction

From ancient to modern times, many philosophers have dealt with the human aspiration that is probably the most desired and, at the same time, the most difficult, if not impossible, to achieve: self-improvement. This topic, from both anthropological and ethical perspectives, has been addressed in classical works: Aristotle’s *Nicomachean Ethics*, Epictetus’s *Manual for Living*, Marcus Aurelius’s *Meditations*, Seneca’s *On the Happy Life*, Augustine of Hippo’s *The Happy Life*, Thomas Aquinas’s *Summa Theologiae I-II*, qq.1–5, Descartes’ *Passions of the Soul*, and Spinoza’s *Ethics*, among others. In one way or another, for these authors, self-improvement implied forging character by practicing virtues and controlling passions (Cfr. McIntyre, 2007).

Today, these questions have made a leap from philosophy to psychology, being treated from different points of view, such as Allport’s (1921, 1927, 1937, 1955) works on personality, Maslow’s (1954, 1962, 1971) theory of self-realization and self-transcendence, Roger’s (1961) model of becoming a person, May’s (1969) existential psychology, Frankl’s (1958) logotherapy, and, more recently, Seligman’s (2002) positive psychology and Wong’s (2011) existential positive psychology, among others.

Likewise, along with academically rigorous essays—such as those by the authors cited, among others—a lot of self-help books designed to modify aspects of character that may result, for oneself and/or others, unpleasant or harmful to health and well-being, have been published and continue to be published incessantly. Many of these publications are superficial literature and offer help that is, in any case, purely cosmetic.

For real, deep, and stable change to take place in some aspect of the character and, with it, in the global configuration of the personal-way-of-being, it is necessary to be willing to carry out a process of realistic self-criticism, self-denial, and coping with difficulties. Ultimately, the modification of character, in this sense, requires cultivating moral virtues, that is, moral habits on which a virtuous character is based (e.g., Thomas Aquinas, 2009).

The Viennese neurologist, psychiatrist, and philosopher Rudolf Allers was one of the principal authors that studied character and contributed to understanding its development and education, including the neurotic character. Before humanistic, existential, and positive psychologies appeared, with their “call” for self-realization and the cultivation of strengths and virtues, Allers dealt with the important matter of what might be called the «essential human calling», that is, the calling to achieve self-perfection. The first editions of *The Psychology of Character* and *Self-Improvement*, Allers’s two works mainly used to elaborate this paper, were published in 1929—in its German edition, *Das Werden der Sittlichen Person*—and 1939, respectively, whereas, for example, Frankl’s *Ein Psychologe erlebt das Konzentrationslager*—*Man’s Search for Meaning* in English editions (e.g., Frankl, 1958)—May’s *The Meaning of Anxiety* and *Man’s Search for Himself*, and Maslow’s *Motivation and Personality* were published in 1946, 1950, 1953, and 1954, respectively.

The aim of the current work is to present Allers’s conception of self-improvement as a process of character education, developing the following ideas: (1) the essence of human existence is self-perfection, that is, self-improvement; (2) egocentricity is the most important obstacle to self-improvement; (3) self-improvement involves self-knowledge; (5) the worst vice impeding self-improvement is pride; (4) self-improvement implies modification—education—of insane aspects of character; (5) a change in character requires effort and more; (6) self-improvement is a personal process of changing toward a better version of oneself.

## Rudolf Allers: A Biographical Sketch

Rudolf Allers, born Rudolf Adeles (Vienna, 1883–1963), was a doctor of Medicine from the universities of Vienna (1906)—where he attended the last course taught by Freud—and Munich (1913). Allers was a strong critic of Freudian psychoanalysis (Allers, 1922, 1941; Cfr. García-Alandete, 2015; Jugnet, 1950). For example, Allers (1941) stated:

If one looks at psychoanalysis it appears to be a wonderful building, in which each detail has its appropriate place, fitting in with every other, and thus evoking the impression of a well-planned and consistent edifice. Nor is this impression deceiving so long as one considers only the façade and the arrangement of the visible architectural elements. (...) But the impression undergoes a profound change if one turns from admiring the façade and the general plan of the building to a closer examination of its foundations. Neither the terrain on which this building is erected nor the way its foundations have been laid can satisfy the exigencies of material soundness and formal correctness. (...)

[…] psychoanalysis rests on several gross logical fallacies, all of which are of the kind known to logic as *petition principii*. Psychoanalysis, in fact more than once, takes for granted what it claims to prove and surreptitiously introduces its preconceived ideas into its reasonings so as to give the impression that these ideas have resulted from facts and evident principles. (p. 33)

Likewise, Allers was Adler’s disciple, and, although he dropped out of his school (Cfr. Echavarría, 2013), he remained close to many Adlerian ideas about character, neurosis, will of power, and will of community—to the extent that Strauss (1943) claimed that Allers was a Catholic Adlerian psychologist.

During World War I, Allers served as a surgeon in the Austrian Army. After the war and until 1927, he worked in the Department of Physiology of the Senses at the University of Vienna. From then and until 1938, Allers was head of the Department of Medical Psychology. Thanks to a personal invitation from the Franciscan friar Agostino Gemelli (1878–1959), who was the founder in 1921 and first Rector of Università Cattolica del Sacro Cuore of Milan, Allers received in 1934 a doctorate in Scholastic philosophy from that university. In 1938, Allers accepted a position as Professor of Psychology offered by the Catholic University of America in Washington. In 1948, he moved to Georgetown University until 1957. In 1960, the American Catholic Philosophical Association awarded him the Cardinal Spellman-Aquinas Medal for his scientific contributions. Allers published numerous works (Allers, 2009b) and lectured at international academic meetings.

Allers’s project, so to speak, was to anthropologically ground psychological science and the pedagogy of character, from a Catholic point of view (Echavarría, 2013; Olaechea, 2016; Seligmann, 2011; Titone, 1957). His psychological observations were based on his own clinical experience, Adler’s individual psychology (Adler, 1912; Allers, 1931), phenomenological and existential philosophies (Allers, 1961)^1^, and, above all, the doctrine of the Fathers of the Catholic Church and medieval Scholastics, particularly Thomas Aquinas (Allers, 1952, 2009a)—whose *De ente et essentia* Allers (1936) translated into German.

## The Essence of Human Existence is Self-Perfection

Allers stated that the essence of human existence is self-perfection, that is, striving for the good or, at least, the love for it. This is “the great motive force driving the world along its path” (Allers, 1939, p. 125). Self-perfection, understood as “the actualization or the becoming really existent of all the qualities originally existing only potentially within the individual” (Allers, 1939, p. 125), is the main good that everyone strives for. Therefore, self-perfection entails self-improvement, which can be understood in a double sense: as an end in itself and as a process. Thus, as an end, self-improvement is equal to self-perfection, and as a process, it is the way self-perfection is gradually actualized in the natural order of being—that is, of being human.

Self-improvement implies having a purpose, not just any purpose, but one that realizes the person’s potential in a correct way. Regarding this, the moral side of human personality acquires particular relevance. The human person is rational, free, and, therefore, responsible for his/her decisions: the individual must choose, consent, and act according to his/her best interests. However, the best does not necessarily coincide with what is pleasant, useful, or beneficial. At this point, Allers coincides with Frankl (1998), who was Allers’s disciple: “The will to meaning is discussed as distinct from the concepts of the will to power and the will to pleasure as they are presented by Adlerian and Freudian psychology, respectively” (Frankl, 1998, p. vii-viii). One has to look beyond oneself, to self-transcend.

According to Allers, character, as “the principle of action” (Allers, 1943, p. 47), is revealed through personal actions related to values, the “general rule of principle of behavior (…) based on the idea one has of the order of values [is] the common principle underlying a man’s actions, which principle refers to values” (Allers, 1939, p. 54).

Changeability of character is the condition for self-improvement. Changing character involves self-knowledge. Therefore, for self-improvement, one has to know him/herself, which is not easy because—among other possible factors—character is a whole structure, so that a small change in one piece alters the whole set (Allers, 1939):

Self-knowledge, as it is needed for self-improvement, has to be more than a knowledge of this or that single imperfection or bad habit or faulty inclination. Character and personality are not built up from single bits as a mosaic is built up from single small stones. (p. 28)

For this reason, it is so important to educate character as a whole through proper practice, but by working on each specific aspect of the character because the whole is, in its totality, unfathomable. A human being is not only an individual, but also, and above all, a person who is “a free and reasonable being that is master, up to a certain extent, of its own nature [and has the] peculiar faculty of being capable of forming and molding himself [which is] the basis of all self-education and self-improvement” (Allers, 1939, p. 30). Likewise, people are different from each other. Therefore, self-improvement is a personal and non-transferable responsibility: “every character-trait, though we have to call it by the same name, is something personal, quite individualized, something peculiar, something personal, to say the least, in every single personality” (Allers, 1939, p. 31).

Character is not seen directly, but rather indirectly, through people’s acts, which rest on certain motives. Knowing these motives is authentic self-knowledge and necessary for self-improvement, that is, to educate character. At this point, a difficulty arises: “In every attempt at self-knowledge, the observer and the observed are one and the same; the actor is at the same time the public. This is the tremendous difficulty and the great danger of self-knowledge” (Allers, 1939, p. 37). In spite of that, self-improvement is still possible.

## Egocentricity, Pride, and Untidy Love for Oneself

For Allers, neurosis is neither a somatic disease nor a mental disorder, but a metaphysical conflict (Allers, 1961):

This problem arises from the awareness –one may call it, if one so wishes, an unconscious one− of man’s essential finiteness and the unwillingness on the part of the individual person to accept the human condition. It is conceivable that the fact of finiteness becomes tolerable, at least to the average person, only when he lives in a real ‘togetherness’ with others, when he exists authentically and, hence, in the mode of ‘being with’. (p. 77)

Egocentricity, pride, and untidy love for oneself are core, one might say pathognomomic, features of neurosis and, therefore, the greatest obstacles to self-improvement, insofar as they imply a “way of escaping from reality and its laws” (Allers, 1939, p. 117). Ultimately, egocentricity, pride, and untidy love for oneself are expressions of mankind’s rebellion against God and the objective order of reality pride (Allers, 1931, 1939, 1943; Cfr. García-Alandete, 2020; Jugnet, 1950).

### Egocentricity

Regarding egocentricity, Allers (1939) stated:

[…] undue concentration on the ego [because of which one] discovers only such features as are immediately related to egoistic ends. The egoist, at least of a certain type, is often rather sharp-sighted for the bad qualities in his fellows, especially for those possibly endangering his own ends, but he is blind to their good qualities, unless they may be used for these same ends. (p. 84)

Allers’s conception of egocentricity, as a nuclear issue of neurotic character, is close to Adler’s definition of neurosis: “Neurosis is the natural logical development of an individual who is (…) filled with a personal, egocentric striving for superiority” (Adler, 1935, p. 9).

Disappointment, ignorance, and error, which are common in the neurotic character, are vices—egoism is the common root of all of them—which were largely addressed by the Fathers of the Church (Cfr. Larchet, 2012) and Saint Thomas Aquinas (2009). Therefore, for self-improvement, the person has to configure his/her character in a way that solves his/her egoistic tendency.

The “normal” character accepts reality and tries to know reality in a rational way. Values, as objective realities, on the one hand, can be known, but, on the other hand, a personal effort should be made to know them. Self-improvement involves knowing values and an effort to realize them, in order to distinguish their relative relevance when two or more of them are in conflict—because there is a hierarchical order of values that is objective and not merely subjective: “Recognizing the objective order of values means that every value is recognized and placed where it belongs” (Allers, 1939, p. 160). However, values not only have to be known, but also realized. In this regard, self-improvement entails a specific way of acting (Allers, 1939):

Acting in a right way means nothing else but regulating one’s actions according to the objective order of values, attempting to realize the highest good among those which can be realized in a given situation. By acting in this fashion, the doer’s personal value becomes greater; this is indeed but an accessory fact; it is not, nor ought it to be, the real motive for action. The value of an action is determined in the first instance not by the subjective moods preceding and accompanying it, but by the objective values it is meant to realize. (p. 134)

Obviously, it is possible to realize values, even for the average person. However, often, certain types of people—neurotics—think this is not true, that one is not capable. This attitude is another serious impediment to self-improvement and, actually, an escape from one's responsibility in realizing values, which requires responsibility and self-transcendence—precisely what the neurotic is not willing to accept. On the other hand, self-improvement is quite different from well-being and/or feeling positive emotions (Allers, 1939):

It is a great mistake to believe that certain mental states or moods are good in themselves, independent of the objects to which they correspond (…) there seems to exist a rather curious glorification of these sentiments independent of the objects to which they correspond. (p. 134)

Precisely, the overvaluation of emotions is, for example, typical of the sentimental character: in this case, reality that is confused with one’s emotions—over knowledge and reason—can make self-improvement difficult^2^ (Allers, 1939):

Action consists in the adoption of the conclusions reason proposes and in realizing the aims resulting from these considerations. The objective truth on which an action is based and the objective values it strives to realize are the only things which matter; feeling is but an accessory factor. (p. 194)

### Pride

Pride is “the first beginning of all downfall” (Allers, 1939, p. 168), that is, the original sin: “The self-willed and self-asserting ego shrinks from the idea of submitting whole-heartedly to the will of God. The devil takes, it is said, the hand when he is offered a finger, but God is sure to take the whole man” (Allers, 1939, p. 182). The neurotic character, therefore, underlies a spiritual, metaphysic phenomenon: the rebellion against the objective order of reality, a rebellion that is characteristic of the neurotic character (Allers, 1939):

Man tends, by his very nature, to please himself and to rebel against the laws of reality and of morals. He is not aware of the fact that this rebellion turns as much against himself as against reality. Man conceives these laws which tend to curb his own stubborn pride as something alien to his person, something outside of the ego, something emanating from a region to which man has no access and to which he does not belong. (…) Man can create for himself the illusion that he is free to acknowledge reality or to reject it.

(…)

By revolting, therefore, against the laws of reality, man wages war against his very own existence; the very personal being of a person becomes endangered by its unwillingness to accept the laws of reality and to submit to them. This is the very reason why maladjustment to reality leads inevitably to conflicts within personality itself. (pp. 207-208)

This characteristic has been pointed out by other psychologists, such as Adler, Horney, and Caruso, among others (Cfr. Alvarez-Segura et al., 2015). This arrogance is “craving for omnipotence. (…) equivalent to making demands where there are not rights” (Allers, 1939, p. 212). Ultimately, therefore, the problem of neurosis is of a spiritual nature in an authentic and real sense for Allers, and not merely metaphorically. In fact, Allers refers frequently to Sacred Scriptures and to the magisterium of the Catholic Church in his writings, not as an intellectual resource, but as a source of truth about the human being and the meaning of his/her existence. Because we all share the same nature, every single one of us is neurotic to some degree, except the saint, who knows himself as a creature and respects the objective order of reality (Allers, 1943):

From the fact that artificiality is an essential component of neurotic behavior, it follows that *the only person who can be entirely free from neurosis is the man whose life is spent in genuine devotion to the natural and supernatural obligations of life*, and who has steadfastly accepted and affirmed his position as a creature and his place in the order of creation; in other words, *beyond the neurotic there stands only the saint*. (p. 346)

Although each of us is “more or less neurotic”—because of original sin—there is an authentic neurotic character, that is, a character that is essentially egoistic and lives in a neurotic, abnormal, perverted, loveless world (Allers, 1961):

Because it is self-centered, the world of the neurotic is essentially a loveless world. (…) in which human relations cannot truly unfold. The neurotic makes demands on his fellows, but he is incapable of complying sincerely with the demands of society. His is not really a “being-with” others (…) into means for an egocentric end. Human beings and things stand almost on the same level; they are more or less usable tools. (p. 77)

Paradoxically, neurotic people believe that they assert and realize themselves by fulfilling their egoistic plans. For a change in the sense of self-improvement to occur, it is necessary to discover the ego self-centeredness that unconsciously keeps us from progressing: “Discovering our hidden egoism is the first condition, if we want to make some progress on the road to perfection” (Allers, 1939, p. 137). It is also necessary to become aware of “excuses”—e.g., one’s genetic inheritance or temperament—which are often used to avoid accepting personal responsibility in the change process because this means leaving behind what gratifies us, although in a false way, typical of an inauthentic life—that is, a neurotic life (Allers, 1943).

A proud person’s existence is dominated by anguish (Allers, 1943). Actually, anguish is a radical fear of existence. Therefore, the neurotic person—who is particularly dominated by pride—is ultimately unhappy because his/her life is inauthentic (Allers, 2008):

The personality of the neurotic is characterized by what German psychologists have called “Unechtheit”, a difficult term to translate. (Approximately: falsehood, falsification). This term is used to designate the divergence between the fundamental and real attitudes of the personality, on the one hand, and the behavior and way of expressing oneself, on the other. The “unecht” (false, apocryphal, imitated, falsified) consists of all the behavior that reflects the role, the mask, the pose; but the “Unechtheit” can go even further, and therefore it becomes a feature of the neurotic personality. (p. 396)

Likewise, in *Existentialism and Psychiatry*, closely following the ideas of existentialist philosophers, Allers (1961) claimed:

Thrown into the world, threatened by the Naught, certain of having to die, shaken, as it were, in the very foundations of his being, man tries to escape facing what he cannot deny. He escapes into an “unauthentic” form of existence, one that is essentially “untrue”, in the sense of true in which we say “true gold”. (p. 44)

### Untidy Love for Oneself

Along with egocentricity and pride, an untidy love for oneself is another core characteristic of the neurotic character, according to Allers. Probably, deep within the neurotic character, there is an atrocious terror of one’s annulment and, hence, the extreme and exclusive self-love of the neurotic, which paradoxically implies isolation from others and from reality (Allers, 1939):

Neurosis is, in fact, what one may call a moral disease; psychology speaks of neurosis as psychogenic, meaning that its causes have to be sought for in mental facts; but it would be quite correct to speak of these troubles as “ethogenic”, meaning that they spring from causes related to morality. (…) extreme egoism is the rule with all cases of neurosis coming under observation. (p. 229)

Perhaps neurosis originates in the early developmental stages, due to an inadequate character education (Allers, 1943). Because of this, the neurotic suffers and is unhappy, although he/she believes the contrary. The neurotic person, for example, can confuse success with happiness and meaning in life. These ideas are close to those formulated by Frankl (1998).

## On the Path to Self-Improvement

### Self-Knowledge

As noted above, for self-improvement to occur, first, one has to be aware of the need for personal change in some aspects of one’s character, particularly regarding personal defects. We usually notice our own defects less than others do, whereas we notice others’ defects more than our own. Self-improvement is not possible if one does not know him/herself (Allers, 1943). Thus, self-knowledge is a prerequisite for changing character (Allers, 1939):

Knowledge of one’s own self is said to be the first step on the way to improvement; we have to know who and what we are first, before finding out whether and in what sense we ought to improve. The most important thing, therefore, is to get a precise idea of one’s own self. (p. 6)

However, knowing oneself is not easy. Many attitudes, convictions, and vices nest within us without our being fully conscious of them. Likewise, we can find ourselves so immersed in the duties of life that it is difficult to achieve a state of interiority that allows us to achieve self-knowledge: “We have to withdraw from all this, to retire into solitude, to seek a peculiar state of mind and congenial surroundings to become capable of discovering the truth about ourselves” (Allers, 1939, p. 24). This idea of drawing on one’s interiority in order to know oneself is taken by Allers from Patristic tradition—e.g., Saint Augustin of Hippo. However, because authentic introspection—that is, without self-deception or self-indulgence—and, therefore, self-knowledge are not easy, help from someone, such as a well-trained therapist, may not only be important, but even essential.

According to Allers, the individual has to start with an objective analysis of his/her own behavior, like a scientist who studies an animal: “And to know exactly what our behavior is like, we have to turn our attention to the effects produced by it in reality” (Allers, 1939, p. 43); that is, what, how, and why a certain action is carried out, especially when that action is a usual way a person acts—that is, when that action is the expression of a habit. The “why” of an action is particularly relevant (Allers, 1939):

It is the aims which determine the quality of an action. An aim is pursued, because the result of its realization is believed to be in some sense “better” than another state of things. Man acts only because he feels the actual state of things to be unsatisfactory and because he hopes that his action will bring about a better state. (p. 46)

### Objective Values

Purposes are directly related to values, that is, to that which is preferable because it is better than another option. Ultimately, acting involves choice and decision. Therefore, acting—one that is conscious and responds to a personal intention and leads to the realization of the purposes—is supported by values: one chooses and acts in accordance with what he/she values as the best—or at least the best option among several alternatives: “Every action aims (…) at the realization of some value; of something which is believed to be better” (Allers, 1939, p. 54). This relationship between purposes and values clearly indicates that the personal act is moral in nature and not merely “existential” in a strictly objective and not merely subjective and circumstantial sense—e.g., it is not, at least not only, a matter of subjective apprehension of the possibilities that life offers *hic et nunc*. Likewise, values are essentially related to the laws of reality, which the neurotic does not accept. At the bottom of neurosis, there is an objective moral problem (Allers, 1939):

Morality is the sum-total of all laws related to the order of values. Values are not vague notions (…) and they are not mere subjective states or feelings. They are a very definite side of reality. (…) Maladjustment to the moral laws is, therefore, as disastrous to personality as is the non-adaptation to what is generally understood by the term of reality. (pp. 208-209)

Nor do values consist of the experience of pleasure (Allers, 1943):

The purpose of an action is the realization of a value, and not of a pleasure. Pleasure can be the goal of an action only because it represents a particular kind of value which has its place in the general scale of values. (…) The pleasure is the reward. (p. 41)

Therefore, if one does not recognize and accept the objective order of reality, he/she cannot advance in the process of self-improvement. This is a task, a duty even, that depends on one’s will and true self-realization: “By revolting against these laws and by making the indeed futile attempt to escape from them, he is undermining the very basis of his own existence” (Allers, 1939, p. 209).

But it is not sufficient to know the objective order of values. The ethics underlying Allers’s self-improvement conception, as depending on the realization of objective values, are not Socratic. It is necessary to develop the right attitude, the personal disposition to act as reason shows to be correct. Ultimately, therefore, it is a matter of awareness of values—which is more than just theoretical knowledge; that is, the synderesis as in Scholastic philosophy, the disposition of practical reason to intuitively apprehend the universal principles of human action—and the will to follow what practical reason shows to be the best. To this, Allers adds reverence, understood as “a peculiar attitude by which we become aware of the dignity of things and of human individuals” (Allers, 1939, p. 210), that is, recognizing their own nature, being respectful of them, and dealing properly with them. Evidently, things are not comparable to people: the former have a price and are useful, whereas the latter have dignity and are intrinsically worthy.

It is necessary for the individual to bow to the objective order of beings and values; it is what his/her mental and moral health consists of. Any rebellion against this order is, in some way, a pathological state that can only lead to some catastrophe. This is what we observe in many cases of neurosis and what we foresee for the people who have been seduced by the lie. (Allers, 1999, pp. 298-299)

The realization of values involves a sort of tension between “is” and “should be”, between actuality and potentiality: “*every action is preceded by a comparison* that (…) is one of *value*”^3^ (Allers, 1943, p. 29). Character is even understood by Allers as being conditioned by an objective realm of values (Allers, 1943):

The character of a man (…) is the justification of his action, something in the nature of a rule or maxim. And inasmuch as this rule embodies the general form of the person’s preference for and rejection of values, character can also be called “the individual categorical imperative”. (pp. 32-33)

The person strives to realize objective values, of which objects are depositaries, and not to feel positive sentiments independently of the objects’ inherent value. Again, these ideas are strikingly reminiscent of Frankl’s in relation to the need to realize values in order to experience the meaning in life.

On the other hand, according to Aristotle (2014) in *Nicomaquean Ethis*, what makes a person happy is developing his/her own nature, that is, his/her rationality. The rationality that is of the most interest in self-improvement is practical rationality, that is, the one that operates in the ethical sphere. Ethics is essentially linked to the practice of virtues, the operating habits that Thomas Aquinas (2009) would say we use to make ourselves better. Practicing virtues is linked to realizing values, which is what a person needs to be happy. Clearly, Allers’ self-improvement model is ultimately based on Aristotelian-Thomistic ethics. Self-improvement is growing toward personal perfection, and, to do so, it is necessary to practice virtues (Allers, 1939):

[…] one must remember the old and fundamental statement of philosophy. Every being strives for the good. […] Among the goods all beings strive for, their own perfection holds a prominent place” (Allers, 1939, p. 125). The aspiration to perfection of a person is nothing other than his perfection as such. This statement seems to be obvious, but it is not whether one thinks about what kind of “thing” a person is and, therefore, what kind of “thing” the perfection of a person is. For Allers, perfection is “the actualization or the becoming really existent of all the qualities originally existing only potentially within the individual. The more perfect a thing becomes, the more visible its very nature becomes. (p. 125)

Will is always free, in spite of conditioning factors beyond one’s will: “we cannot discover their influence but by a process of exclusion, determining first the role played by will” (Allers, 1939, p. 48). Accordingly, a person is ultimately self-determining. Even habits are modifiable by will (Allers, 1939):

The development of a habit is not a mere mechanical process; even for a habit to become established some kind of consent of the person is necessary. This consent may simply consist in a non-resistance; but whether taking the form of an actual assent or a mere letting things go, it is always an act of will. A habit already developed is not at all beyond the control of will. (p. 79)

Complete self-knowledge is not possible, and the self-knowledge process is not without its dangers, which can even strengthen egocentricity instead of fighting against it (Allers, 1939):

This search for the truth about ourselves and the attempt to unveil the hidden egoistical tendencies have some dangers of their own. They too may become masks of egoism and vanity. Instead of pursuing his task for the sake of truth and an objective good −with the perfection of personality being such a good− man may go on hunting for faults merely to gratify his vanity or to find some plausible excuse for not making progress. (p. 199)

Despite these difficulties, it is possible to change one’s character.

### The Will to Change

The idea that character is immutable does not arise from empirical data, but rather from philosophical convictions. On the contrary, it is an empirical fact that character can be changed. Moreover, character can be educated, and there is an optimistic point of view about the possibility of changing one’s bad habits or personal features. According to Allers, “we can change (…) personality and character may change and that this change can be brought about by our own doing (…) [but it demands strength of will, which] depends very much on the goals proposed to the will” (Allers, 1939, pp. 6 and 13).

If people do not have goals, it is difficult for them to feel motivated to change the aspects of their character they think they need to change. Moreover, in addition to possible psychological factors such as fear or vanity, habit can play an important role in the difficulty of acting as one desires, although even the most ingrained habits can be modified if one has powerful reasons for it, reasons that have to be discovered (Allers, 1939):

A man, therefore, who feels it impossible not to do certain things, needs to have some reason for doing them, even though he may not be aware of this fact. These reasons have to be discovered, or no attempt to get rid of the habit will prove successful. (p. 22)

In the foregoing lines, we stated, according to Allers, that self-knowledge is a condition for self-improvement. However, it is not sufficient: one has to establish the pathway to self-transformation and translate intention into action, which is not easy. For example, many people claim that they cannot change their character because of their weak will. However, weakness of will is actually an excuse to avoid facing the challenge and effort of character change (Allers, 1939):

Experience shows that changes in character occur and that they may be brought about by natural influences. Among these influences, man’s own will and endeavor play quite a prominent part.

(…)

The idea of being incapable of any change is itself part of the character that is in need of change. (…) changing is a difficult and painful task; and human nature tends to escape, as fast as possible, all unpleasantness. (…) it is just this conviction of not being able to change which ought to supply a strong reason for attempting to do so. (…) If human character were not susceptible to very great changes, it would be very strange indeed that so many of today’s psychologists, pedagogists, and philosophers deal in their writings with the problem of character education. (pp. 9-10)

Will and action are inseparable, to the point that both are like two sides of a coin: “will and execution are so merged one into the other (…) willing − real willing − and doing are but two sides of one and the same human act” (Allers, 1939, pp. 14–15). However, the determination to act as desired depends on the presence of significant personal goals: “what is called weakness of will is due not so much to lack of energy as to lack of unity of the will. The trouble lies more with purpose than with will” (1939, p. 15). Thus, it is very important to distinguish between mere knowledge and authentic motivation. It is not enough to know what I have to do and what I want to do, but it is also necessary to act. In order to act—in a certain manner, actually, in order to do better, what ultimately motivates us to action? Are values what motivate us to act, in order to do better: “Knowledge of what is objectively better or of a higher value is indeed not a sufficient motive for action” (Allers, 1939, p. 16). Therefore, between will, act, and values, there is an intrinsic relationship.

However, many people are convinced that they cannot act as they wish, probably because they have never attempted to do what they believe they want or have to do, or because they have attempted to do so only a few times, but not every time it was necessary. Likewise, many people “know that they have this or that undesirable quality or habit, and they would be quite glad to get rid of it; but they believe this to be impossible, because this quality is, as they say, part of their nature” (Allers, 1939, p. 49). However, it is not true: it is possible to change one’s character. This is the principle of acquiring a virtuous character: to practice virtues over and over again. In some cases, actually, it is not that one cannot, it is that one does not want to.

There are people who try to evade their existential responsibility for self-perfection by making excuses; for many of them, excusing their responsibility is even a habit that “springs mostly from vanity, from the desire to avoid the unpleasant feeling of having made a mistake or committed a fault [because] Man wants to appear better than he is in the eyes of the world, but even more in his own” (Allers, 1939, p. 173). This attitude reveals a «lack of sincerity», that is, the “conviction of not being capable of real progress” (Allers, 1939, p. 175). Precisely, the first step toward self-improvement is to recognize that one is not perfect and, therefore, that it is necessary to make an effort to improve in order to achieve self-perfection. In other words, one must feel dissatisfied with him/herself and aspire to be better, although with humility and prudence in judging what has to be changed (Allers, 1939):

Dissatisfaction with himself is the strongest motive starting him on the road to perfection. But this feeling is reasonable and valuable only so long as it deals with sides of reality which are essentially capable of being changed, and it becomes nonsensical when turning on features of reality which must necessarily remain the same. (p. 178)

Moreover, due to personal character flaws and limitations, self-knowledge may be objectively difficult, and so the effort it takes can cause tiredness and disappointment. This effort is essentially a moral effort, by practicing certain virtues: humility, constancy, trust, sincerity, and patience, among others, and “when starting on the way of progress and improvement, we have to be prepared for a lengthy period of unceasing endeavor, for plenty of relapses and failures. But there is, painful as this need is, one great compensation” (Allers, 1939, p. 205), and, overall, attempts to achieve self-improvement are meaningful.

First and foremost, one must develop love toward his/her neighbors. That is, one has to cultivate the «will to community», a primal human tendency: “the moral goal of the will to community is love, love of one’s neighbor and every other kind of love” (Allers, 1943, p. 122). Just like the law of the «will to power» is “self-preservation, the development of the sense of personal value, and the complete realization of himself by the individual” (Allers, 1943, p. 122), the law of the «will to community» is love. The «will to community» operates as a counterweight to an excessive «will to power». Why would one love his/her neighbors? Because love is the opposite of egocentricity, and therefore it is “the very key which opens the gate barring the way to perfection. Egoism is best uprooted (…) by the strengthening of the conviction that it is utterly nonsensical” (Allers, 1939, p. 227). Particularly, the neurotic person has to stop loving him/herself untidily and start loving others (Allers, 1943):

Only in living connection with others will he find succor in his isolation and abandonment in this great and terrible world; only in the consciousness of being one amongst others can he free himself from the oppression created by his individual impotence, which impedes his will to self-assertion, and so become capable of a voluntary subordination to that which simply is and prevails. (p. 133)

At these points, Allers closely follows Adler’s (1931/1998) theory:

There have always been men who understood this fact; who knew that the meaning of life is to be interested in the whole of mankind and who tried to develop social interest and love. (p. 9)

### Self-Transcendence

One has to “recognize these laws [of objective reality] as ruling over all things and over every individual as well”, in a way that will lead to discovering that “our nature is a rather limited one, that it is shut up within definite borders. (…) Each of us has to recognize the limits set to him and his activity” (Allers, 1939, p. 230). That is, none of us is imperfect, limited, vulnerable, fallible, and mortal. In other words: none of us is God, but rather we are mere creatures. Humility emerges, in connection with this, as the essential virtue that connects the person with reality.

Escaping from personal responsibility for acting, for example, by developing an illness, is a symptom of neurosis, which makes the person incapable of leading a functional and positive daily life, according to Allers. Moreover, as mentioned above, neurosis is equal to egocentricity, an egoistic attitude that would mean that the person cannot afford to make a mistake that concerns him/her personally. In relation to that, Allers’s ideas are very close to those of Adler (1931/1998), who stated about neurotics:

It is especially frequent amongst neurotics that these two problems of society and love are the problems that they try to evade. They make no approach to the other sex or they make wrong approaches. They have no friends and they do not interest themselves in other people. But they are occupied day and night with their business. They think of it and dream of it in bed. They throw themselves into a tension; and in their tension the neurotic symptom appears; stomach irritation or some such trouble. They feel now that their stomach trouble excuses them from meeting the problems of society and love. In other cases the man is always changing his occupation. He can always think of an occupation which would suit him better. In the end it appears that he is not occupied at all; he is always vacillating from one thing to another. (pp. 154-155)

This egotistical attitude of neurotic people is radically opposed to the objective order of values, which are the aim of personal acting in order to achieve self-improvement. Therefore, egocentricity is also equivalent to axiological subjectivism (Allers, 1939):

Goodness or value, the goal every striving is aiming at, is primarily an objective quality of things or events. To man is given the task not of creating values or of attributing them to things originally indifferent as to value, but the task of discovering values existing in the world wherein he lives and of becoming aware of values as yet not real which he has to bring into existence.

Acting in a right way means nothing else but regulating one’s actions according to the objective order of values, attempting to realize the highest good among those which can be realized in a given situation. By acting in this fashion, the doer’s personal value becomes greater; this is indeed but an accessory fact; it is not, nor ought it to be, the real motive of action. The value of an action is determined in the first instance not by the subjective moods preceding and accompanying it, but by the objective values it is meant to realize. (pp. 133-134)

According to Allers, values are objective, not merely subjective; if values were merely subjective, a person would be closed in on himself, and that is the neurotic’s main characteristic. Neurotic people are prevented from improving themselves because they are locked in their egoistic world—e.g., their dreams, desires, fears, and anxieties, among others—(Allers, 1931, 1939, 1943), which is, in some way, a rebellion against reality—the objectively limited human nature and life’s duties. The neurotic’s bad habits—a more appropriate term comes from classical moral philosophy: vices, such as vanity, inordinate ambition, laziness, among others—have one and the same root, according to Allers (1939):

They are all due to a disturbance of the true equilibrium between reality and the ego, objectivity and subjectivity. (…) It is always the ego which pushes, as it were, into the foreground, which attempts to occupy a place it is not entitled to; it is subjectivity trying to get the upper hand over reality. These attempts are bound to fail. Reality is stronger than the ego ever can hope to be. These attempts turn, moreover, against the ego itself; disregarding reality amounts to imperiling the ego too. (p. 119)

For self-improvement, one has to accept and commit to reality. Thus, unrealistic self-improvement is not possible. Nevertheless, the neurotic person believes that he/she asserts and realizes him/herself by fulfilling his/her egoistic plans. Fortunately, the neurotic character can be changed, cured of its main evil: egocentricity.

When a person is not capable of self-transcending his/her ego, he/she becomes a selfish person who cannot realize the meaning of his/her life. For Frankl too, egocentricity is equal to neurosis: a noogenic neurosis, that is, a neurosis due to existential meaningless (Frankl, 1998):

The essentially self-transcendent quality of human existence renders man a being reaching out beyond himself. (p. 20)

Most important (…) are those noogenic neuroses which result from the frustration of the will to meaning, from what I have called existential frustration, or from the existential vacuum. (p. 39)

Human beings are transcending themselves toward meanings which are something other than themselves, which are more than mere expressions of their selves, more than mere projections of these selves. Meanings are discovered but not invented. (p. 72)

Maslow also stated that self-transcendence is a necessary condition for full self-realization, understood as one’s liberation from egocentricity from a transpersonal point of view, beyond self-realization—that is, beyond fulfilling one’s potential. Self-transcendence would be the highest level in the personal process of growing as a person; one who is not capable of self-transcendence remains egocentrically closed in on him/herself (Maslow, 1971):

Transcendence refers to the very highest and most inclusive or holistic levels of human consciousness, behaving and relating, as ends rather than means, to oneself, to significant others, to human beings in general, to other species, to nature, and to the cosmos. (p. 269)

Reaffirming these ideas, Frankl (1975) responded critically to Maslow’s motivational principle of self-realization:

[…] the real aim of human existence cannot be found in what is called self-actualization. Human existence is essentially self-transcendence rather than self-actualization. Self-actualization is not a possible aim at all for the simple reason that the more a man would strive for it, the more he would miss it. For only to the extent to which man commits himself to the fulfillment of his life’s meaning, to this extent he also actualizes himself. In other words, self-actualization cannot be attained if it is made an end in itself, but only as a side-effect of self-transcendence. (p. 175)

## Self-Improvement and Personal Ideal

Self-improvement involves changes in character, and these changes are ultimately one’s movement toward a better version of oneself, that is, toward a personal ideal (Allers, 1943):

[…] those pictures fashioned by man of what he should be and how he ought to act; (…) the ideal, regarded as a directional force and as a goal, is somehow experienced through the difference that is felt to exist between it (the ideal) and the person as he really is. (p. 190)

There is a difference between the person one is and the person one would like to be, and one has to perceive this difference as acceptable so that the change—and therefore the self-improvement—advances from the possible to the real. What does this attitude depend on? The rule by which change of character—that is, the self-improvement process—should be regulated is “the complete realization of all the positive potentialities inherent in the person” (Allers, 1943, p. 207), which points to the realization of values, that is, of what is personally worthy in order to achieve self-perfection. Just like Aristotelian Scholasticism philosophy teaches that the act is the perfection of what is potential (Cfr. Aquinas, 2009), self-perfection can be understood as the actualization of the person as this person. In this regard, one’s entire life is a constant effort for self-improvement, in the indicated sense of the realization of values, despite the fallen state of human nature. This is the meaning of human existence (Allers, 1943):

Individual life (…) is nothing more than the successive realization of all values inherent as potentialities. The transformation of potentiality into act (…) is the essence and meaning of human life. I am convinced that the tension between what has been realized and what remains to be realized (…) provides the real motor power, the actual driving-force, by which the movement of life is maintained. When a man has ceaselessly realized all that there was of value-potentialities in the depths of his being, his life must come to a standstill; he must die. That is, I think, why so many saints die young. (p. 210)

Because one should not aspire to any ideal except the one that best fits his/her natural personality and circumstances, the individual’s free choice and autonomy are not absolute, but they must submit to the best. Furthermore, “man may in no wise be set up as something absolute or be regarded as an end in himself” (Allers, 1943, p. 214). Paradoxically, self-perfection is reached by the person who does not try to achieve it directly, but rather through the realization of values, of the ultimate perfection: “many goals − including the ideal of perfection − are unattainable if directly striven for. Rather does perfection come to him who does not seek it (and is thus not self-seeking) but seeks that which is perfect” (Allers, 1943, p. 236). In this regard, as stated above, the role of education in the process of self-improvement is irreplaceable, fostering the development of the person through the realization of those values that are more in line with his/her nature (Allers, 1943):

In education, we are faced with the difficult task of steering a middle course between all those measures which tend to weaken the experience of incorporating a personal value (legitimate self-esteem), and those which are calculated to lead to an “absolutizing” of the self. (p. 239)

Ultimately, Allers considers that the Catholic way of life, so to speak, perfectly represents the ideal to which the person should aspire (Allers, 1943):

[…] the character ideal that can alone fully satisfy the conditions of man’s being must (…) reconcile and synthesize the conflicting claims of the individual and the community, of the value of the individual and the value-giving totality, of the finiteness of the creature and the call to participation in the divine life. (…) Catholic life, based on Catholic principles, can reconcile the antitheses of our being and bring about a resolution of tension. (pp. 239-240)

It is evident, therefore, that for Allers the education that will most promote self-improvement is the one based on Catholic doctrine. Likewise, given that the subject of that education is the person, both the adequate anthropology and characteriology on which such educational action should be based would have to be theological (Allers, 1943):

[…] *theoretical characteriology must be founded upon a theory of values and ultimately, therefore, upon ontology and metaphysics*. A working theory of character, therefore, needs the constant support of ethics, which is the science of the realization of values; thus, characteriology itself must refrain from determining values, but cannot exist without paying regard to them. The further implication is that since, for every one of us, “all that there is of value can only culminate in, as it originally arose out of, a *summum bonum, the science of characteriology must ultimately be bound up with religion*. A naturalistic system of characteriology is inherently impossible. (p. 60)

Ultimately, sanctity is the most advanced degree of self-perfection one can achieve, the best version of oneself (Allers, 1943). In fact, for Allers, the saint is someone who lives authentically, recognizing the objective order of reality and his/her place in the cosmos as a limited, fallible, vulnerable, and ultimately mortal creature. Sanctity is, therefore, beyond neurosis (Cfr. Jugnet, 1950), because the saint recognizes that he/she is not God but a mere creature. Therefore, self-improvement involves humility as the cardinal virtue opposite to pride.

## Conclusions

According to Allers, the main purpose of one’s existence is to achieve self-perfection. This goal involves fighting against the tendency of the human being, that is, egocentricity, pride, and untidy love for oneself. These vices or character flaws are essentially metaphysical in nature and a consequence of original sin. The struggle for self-perfection consists of perceiving objective reality, fundamentally in the realm of values, and educating one’s character through the practice of virtues. Although the foundations of Allers’s ideas lie essentially in the Catholic doctrine, they are shared in their essence by other authors, mainly Adler regarding the characteristics of neurotic character, and Maslow, Frankl, and existential positive psychology regarding self-transcendence by realizing values. To some extent, Allers’s ideas are shared by positive psychology, with its emphasis on the need to cultivate virtues to achieve happiness.

For Allers, there is an essential relationship between character, ethics, and metaphysics. In other words, for Allers, character has an essential ethical-metaphysical nature, and not merely psychological. Due to his Aristotelian Thomistic approach to understanding human nature, Allers considered it necessary and adequate to consider character, and not personality, as the nucleus from which the person configures the way he/she is-and-acts in the world (Allers, 1943). His conception of character intrinsically involves the moral and religious dimensions of human nature. Thus, Allers’s approach to character and self-improvement can be conceived as psycho-ethical-metaphysical.

Currently, Allers’s conception of character and, in general, the character approach in psychology and psychiatry are not contemporary, either theoretically or clinically. Particularly, Allers’s metaphysical and *a fortiori* religious approach is for the psychological sciences part of the past, as is, on the other hand, the concept of neurosis. As is known, the term «neurosis» was replaced with the term «disorder» by the American Psychiatric Association (APA, 1980) and the World Health Organization (WHO, 2015). However, it continues to be a historically interesting approach, and it is still under study today (Banicki, 2017). Moreover, the classical doctrine of neurosis as character flaws could be of interest for better understanding certain mental disorders from both phenomenological and existential approaches—e.g., personality disorders (Alvarez-Segura et al., 2015)—complementing the currently dominant medical perspective—as in the not so distant past, e.g., Binswanger (1958). Particularly, Allers’s conception of character—its theory, pathology, and therapy—insofar as it is based on Catholic doctrine, could be interesting for a Christian approach to mental health sciences, both theoretically and clinically (e.g., McMinn & Campbell, 2007; Severson, 2013).
